# Adherence to a web-based intervention program for traumatized persons in mainland China

**DOI:** 10.3402/ejpt.v5.26526

**Published:** 2014-12-09

**Authors:** Zhiyun Wang

**Affiliations:** Department of Psychology, Wuhan University, Wuhan, People's Republic of China

**Keywords:** e-Health, online interventions, web-based, Chinese My Trauma Recovery, adherence, depression, PTSD, psychotrauma, self-efficacy

## Abstract

**Background:**

This paper investigated adherence to a self-help web-based intervention for PTSD (Chinese My Trauma Recovery, CMTR) in mainland China and evaluated the association between adherence measures and potential predictors, for example, traumatic symptoms and self-efficacy.

**Methods:**

Data from 56 urban and 90 rural trauma survivors were reported who used at least one of the seven recovery modules of CMTR.

**Results:**

The results showed that 80% urban users visited CMTR four or less days and 87% rural users visited CMTR for 5 or 6 days. On average, urban users visited 2.54 (SD=1.99) modules on the first visiting day and less from the second day; rural users visited 1.10 (SD=0.54) modules on the first visiting day, and it became stable in the following days. In both samples, depression scores at pre-test were significantly or trend significantly associated with the number of visited web pages in the relaxation and professional help modules (*r*=0.20–0.26, all *p*<0.14); traumatic symptom scores at pre-test significantly or trend significantly correlated to the number of visited web pages in the relaxation, professional help, and mastery tools modules (*r*=0.20–0.26, all *p*<0.10). Moreover, urban users’ coping self-efficacy scores at pre-test significantly or trend significantly related to the number of visited web pages in the relaxation, professional help, social support, and mastery tool modules (*r*=0.20–0.33, all *p*<0.16).

**Conclusions:**

These findings suggest that individuals tend to focus on one or two recovery modules when they visit CMTR, and the number of web pages visited during the intervention period relates to users’ traumatic and depressive symptoms and self-efficacy before intervention.

